# Standardised patients with intellectual disabilities in training tomorrow’s doctors

**DOI:** 10.1192/pb.bp.113.043547

**Published:** 2014-06

**Authors:** Bini Thomas, Ken Courtenay, Angela Hassiotis, Andre Strydom, Khadija Rantell

**Affiliations:** 1 North East London NHS Foundation Trust; 2 Barnet, Enfield and Haringey Mental Health NHS Trust; 3 Mental Health Research Unit, University College London; 4 Camden and Islington NHS Foundation Trust; 5 Joint Research Office, University College London

## Abstract

**Aims and method** To develop a programme to help undergraduate medical students and postgraduate trainees to improve their skills in communicating with people with intellectual disabilities through teaching sessions that had input from simulated patients with intellectual disabilities. We conducted four sessions of training for 47 undergraduate 4th-year medical students. The training involved a multiprofessional taught session followed by a clinical scenario role-play with simulated patients who were people with intellectual disabilities. The training was assessed by completing the healthcare provider questionnaire before and after the training.

**Results** There were improvements in the students’ perceived skill, comfort and the type of clinical approach across all three scenarios.

**Clinical implications** By involving people with intellectual disabilities in training medical students there has been a significant improvement in students’ communication skills in areas of perceived skills, comfort and type of clinical approach which will raise the quality of care provided by them in the future.

A large proportion of people with intellectual disabilities have a comorbid diagnosis of cerebral palsy, epilepsy, hip dislocation, chest infections, eating and swallowing problems, gastro-oesophageal reflux, constipation, incontinence, osteoporosis and mental illness.^1^ In 2008, an independent inquiry into access to healthcare for people with intellectual disabilities in the UK reported that they have more unmet needs and receive less effective treatment from the National Health Service (NHS).^2^ The inquiry reported that people with intellectual disabilities find it much harder to access treatment for general health conditions due to failure in making reasonable adjustments and poor communication between the patient, their carers and healthcare professionals and called for better training for all staff in the NHS.

*Healthcare for All*,^1^ a report from the Department of Health published in response to preventable deaths of people with intellectual disabilities in acute hospital care in England, recommended that those with responsibility for the provision and regulation of undergraduate and postgraduate clinical training must ensure that curricula include mandatory training in intellectual disabilities. In 2010, the General Medical Council (GMC) included the need to improve doctors’ understanding of the needs of people with intellectual disabilities in their 3-year plan to improve care in the UK. A consultation document on the GMC’s equality scheme for 2011-2014 states its intention to develop materials to raise doctors’ awareness of the needs and experiences of people with intellectual disabilities.^3^

## Using simulated patients in medical training

Simulation in medical education dates back to the 16th century, when mannequins were developed to teach obstetric skills to reduce maternal and infant mortality rates.^4^ The purpose of involving standardised patients in training medical students is for the medical students to be able to confidently treat patients with disabilities and understand the specific disability-related issues.^5^ Despite the ethical tensions and practical dilemmas associated with involving real patients as standardised patients in training programmes, simulation-based medical education is an important tool in the safe delivery of medical care.^6^ The advantages of involving patients include achieving consistency within the examination^7^ and the provision of constructive feedback to the students by actors.^8^

In US universities, a variety of educational programmes have been conducted aiming at providing students with opportunities to gain experience with patients with disabilities and to enhance students’ interview skills.^5^ In 1999, a telephone questionnaire was administered to staff of Australian medical schools to determine the amount and nature of undergraduate teaching on the healthcare of people with intellectual disabilities provided to students. Based on the consultation and expert advice, it was recommended that the curriculum should focus on the three key areas of attitude, skills and knowledge.^9^

St George’s Hospital Medical School in London has led the way in the UK in involving people with intellectual disabilities in undergraduate teaching.^10^ The students reported that training improved their understanding of non-verbal cues and helped them develop more thoughtful and thorough approaches to circumvent diagnostic over-shadowing.^11^ At University College London (UCL), we aimed to develop a programme to help undergraduate medical students and postgraduate trainees to improve their skills in communicating with people with intellectual disabilities by involving them as standardised patients in the teaching sessions. The project was a joint venture between UCL, University College London Hospitals NHS Foundation Trust, Camden and Islington NHS Foundation Trust, and Barnet, Enfield and Haringey Mental Health NHS Trust. UCL funded the project with a grant from the Executive Subcommittee on Innovation in Teaching, Learning and Assessment (ESCILTA) to train and employ individuals with intellectual disabilities as simulated patients.

We sought advice from the UCL research ethics board, who confirmed that ethics approval was not needed for the teaching project.

## Method

We worked with Access Simulations (www.access-simulations.co.uk), a group of four actors with intellectual disabilities, to carry out a pilot of people with intellectual disabilities in the role of standardised patients. Twenty-one individuals from local boroughs interested in training as standardised patients attended the pilot session where they met the four actors. They observed a practical scenario played out through a video link and were given opportunities to meet the actors and to discuss in detail the advantages and challenges of being involved in training.

### Recruitment of actors

Twenty-one candidates responded to an advertisement on standardised patient training. All attended an interview during which they were asked to carry out a performance task, for example buying a newspaper. We selected nine individuals who received training for 8 weeks. A support worker was recruited from the local Advocacy Project (www.advocacyproject.org.uk) to facilitate the sessions.

Each person was trained in four clinical scenarios depicting common clinical situations: simulating a person with depression; requesting an explanation of a medical procedure or medication; receiving bad news; and taking history from a person with epilepsy. The aim of the training was for the actors to participate as standardised patients in role-play stations and in medical student examinations. Each clinical scenario was written by a specialist trainee in intellectual disability psychiatry (B.T.). Scenarios were discussed in detail with all participants to ensure that they understood them and became familiar with the tasks required within the scenario. The patients were also trained to give structured feedback to the students after each role-play station.

All steps were taken to ensure training materials were in accessible format. We obtained written informed consent from the patients to participate in the training and to be video recorded.

### Training in medical consultation with patients with communication difficulties

We proceeded to conduct a pilot of four sessions of training for undergraduate 4th-year medical students at UCL Medical School at the end of two educational blocks on neurosciences. The students were offered places on a first-come, first-served basis. Forty-seven participants in total attended the course. All participants had attended a 3-hour lecture on intellectual disabilities as part of their neurosciences curriculum and approximately nine students per block had a 3-week clinical placement in a community intellectual disability service run by two authors (A.H. and A.S.).

The training consisted of a morning session by a speech and language therapist, which involved didactic teaching, group work, watching a communication DVD, and basic Makaton training (a simplified sign language based on British Sign Language). After the taught session, students were divided into four groups of four and rotated through four stations. All students had at least one opportunity to interact with a patient with intellectual disabilities. Following each station, the actors and facilitators gave the students structured feedback. At the end of the training session the students received final debriefing chaired by a consultant psychiatrist in intellectual disabilities (A.S., A.H. or K.C.). All attendees were given a certificate of attendance for the course that could be included in their portfolio.

### Instruments

We undertook a before-and-after training evaluation using the healthcare provider questionnaire, which is designed to explore healthcare professionals’ feelings in different situations.^12^ The participants were presented with three scenarios. The first one involved seeing an out-patient with a common complaint, but with no physical or intellectual disability. The second scenario involved assessing a patient with mild intellectual disability with a similar complaint. The third scenario involved assessing a patient with severe intellectual disability with the same health problem as in the other two scenarios. The students were asked to make a series of self-attributions along 12 items presented in a 7-point semantic differential format and representing three dimensions:

1. perceived skill (skilled/unskilled, efficient/inefficient, capable/not capable, comprehending/guessing)
2. comfort (comfortable/uncomfortable, calm/anxious, graceful/awkward, confident/apprehensive)
3. type of clinical approach (rational/intuitive, intellectual/instinctive, sophisticated/naive, objective/subjective).

The primary hypothesis was that the course using standardised patients with intellectual disabilities would improve medical students’ perceived skill, comfort and type of clinical approach in the scenarios with people with mild and severe intellectual disabilities. We were interested in testing the hypothesis that improvements would be more pronounced in the scenario with the person with severe intellectual disability. We made adjustments for demographic factors such as gender, age, student’s country of birth, and previous exposure to people with intellectual disability.

### Statistical analysis

This was an exploratory study and no prior sample size calculation was carried out. Continuous data were described using mean and standard deviation or median and interquartile range depending on data distribution. Categorical data were summarised using count and percentages. The pre- and post-training scores were compared using the paired *t*-test of its non-parametric equivalent for each of the three scenarios in turn. Results were not adjusted for multiple testing. Analysis of covariance (ANCOVA) was carried out with each of the post-training scores as the outcome and the pre-score disability groups (mild and severe), and other patient characteristics as covariates (age, gender and ethnic group), with robust standard error. Analyses were carried out in Stata V11 on Windows Vista Ultimate.

Multiple regression analysis was carried out to account for any confounding factors that included pre-training scores, age, gender and ethnicity.

## Results

The mean (s.d.) age of the participants was 23.2 (1.6) years; 57% (*n* = 27) were female and 82.9% (*n* = 39) were born in the UK. Twenty-five participants (53.2%) had entered British as their ethnic background, 29.8% (*n* = 14) were British Asian and 17.02% (*n* = 8) were from other ethnic groups. The majority of participants (89.4%, *n* = 43) had had previous contact with people with intellectual disabilities at school, during voluntary work or in the family. Almost all (93.6%, *n* = 44) had additional qualifications in various specialties.

The differences in before and after questionnaire scores are presented in Table 1. The table shows that there was a significant decrease in the post-score for all factors, in particular larger differences between the pre- and post-measurement scores are noted for individuals with severe and mild intellectual disability.

**Table 1:** Summary of differences between the pre- and post-training scores for each factor by patient disability level scenarios

Patient disability level	Factors	Pre-training scores Mean (s.d.)	Post-training scores Mean (s.d.)	Mean difference (95% CI)	*P*
None					
	A. Perceived skills	12.98 (4.35)	11.55 (3.82)	1.43 (0.50-2.35)	0.002^*^
	B. Comfort	9.87 (3.74)	8.72 (3.13)	1.15 (0.37-1.92)	0.004^*^
	C. Type of clinical approach	10.87 (3.71)	9.68 (2.97)	1.19 (0.42-1.96)	0.003^*^
					
Mild					
	A. Perceived skills	18.51 (4.63)	12.04 (3.60)	6.47 (5.27-7.67)	<0.001
	B. Comfort	14.43 (3.69)	9.30 (2.96)	5.13 (4.28-5.98)	<0.001
	C. Type of clinical approach	13.85 (3.45)	10.09 (3.28)	3.77 (2.69-4.84)	<0.001
					
Severe					
	A. Perceived skills	23.21 (5.26)	14.34 (4.16)	8.87 (7.49-10.25)	<0.001
	B. Comfort	18.28 (4.23)	10.96 (3.51)	7.32 (6.18-8.46)	<0.001
	C. Type of clinical approach	16.96 (4.39)	11.48 (3.35)	5.48 (4.12-6.84)	<0.001

^*^ *P*-value obtained using Wilcoxon test. The median difference for perceived skills and comfort are –1 (95% CI –1.7 to 0) and 0 (95% CI –1 to 0) respectively.

### Perceived skills (factor A)

There was a significant improvement in the students’ perceived skill in managing patients with none, mild or severe disability post-training. The corresponding mean differences were: 1.43 (95% CI 0.50-2.35; *t*(46) = 3.14, *P* = 0.002), 6.47 (95% CI 5.27-7.67; *t*(46) = 10.82, *P*<0.001) and 8.87 (95% CI 7.49-10.2; *t*(46) = 12.96, *P*<0.001) respectively.

### Comfort (factor B)

This factor reflected the level of comfort experienced by medical students while interacting with people with varying levels of intellectual disability. When tested for difference in the pre- and post-training scores there was significant improvement in the scores in communicating with people with no disability (1.15 (95% CI 0.37-1.92), *t*(46) = 2.98, *P*<0.005), people with mild intellectual disability (5.13 (95% CI 4.28-5.98), *t*(46) = 12.14, *P*<0.001) and people with severe intellectual disabilities (7.32 (95% CI 6.18-8.46), *t*(46) = 12.91, *P*<0.001) post-training.

### Type of clinical approach (factor C)

There was a significant improvement in the type of clinical approach adopted by students in managing patients with none, mild or severe disability post-training. The corresponding mean differences were: 1.19 (95% CI 0.42-1.96, *t*(46) = 3.12, *P*<0.005), 3.77 (95% CI 2.69-4.84, *t*(46) = 7.05, *P*<0.001) and 5.48 (95% CI 4.12-6.84, *t*(46) = 8.13, *P*<0.001) respectively.

The relationships between score (pre-/post-) and the scenarios remained statistically significant throughout even after adjustment for demographic factors.

Table 2 shows the effect of training on communicating with patients with mild *v*. those with severe disability. The mean scores for the severe disability scenario were significantly higher compared with the mild disability scenario based on the adjusted and unadjusted analyses in all factors considered, indicating that the impact of the training was much higher in terms of managing patients with severe intellectual disabilities.

**Table 2:** Comparing effect of the training in communicating with people with mild intellectual disabilities *v.* severe intellectual disabilities

	Unadjusted analysis		Adjusted analysis	
Factor	Estimates (95% CI)	*P*	Estimates (95% CI)	*P*
A. Perceived skills	2.40 (1.22-3.59)	<0.001	2.40 (1.21-3.60)	<0.001
B. Comfort	2.19 (1.31-3.07)	<0.001	2.19 (1.30-3.08)	<0.001
C. Type of clinical approach	1.71 (0.43-2.99)	0.01	1.72 (0.43-3.01)	0.01

*P*-values obtained using linear regression adjusted for cluster affect. Analysis was adjusted for pre-training scores, age, gender and ethnicity.

## Discussion

We have shown consistent improvements along all three factors (perceived skill, comfort and type of clinical approach) after the training. However, the improvement along the dimensions was more significant for scenarios involving people with severe intellectual disabilities compared with those involving people with mild intellectual disabilities.

### Feedback from students

Feedback from students was positive, with the majority rating the presentations, role-plays and debriefing as excellent. The students commented that the training had encouraged them to interact with the patients and to pay special attention to non-verbal communication. They also gained more confidence in using a degree of sign language, pictures and drawings as communication tools to develop a good rapport with the patient. Students suggested making the training compulsory for all medical students rather than it being an optional course. They appreciated the involvement of patients in the role-play scenarios.

### Strengths and limitations

The actors with intellectual disabilities were actively involved throughout the process of designing the role-play stations for the training programme and in providing feedback to the medical students. Special care was taken to ensure the scenarios demonstrated general clinical situations rather than specifically focusing on mental health to enhance the applicability of the training to all clinical scenarios that doctors would face in their everyday practice. These ‘standardised patients’ played a major role in providing valuable individual feedback to the students, which was highly appreciated by all participants.

However, we could only offer training to a limited number of medical undergraduates in this pilot project. As candidates were offered a place on a first-come, first-served basis, there is a possibility that the students who responded first were especially motivated and therefore more willing to engage with the training and to use it in a positive manner. This could lead to volunteer bias, which might have had an effect on the scores.

### Patient perspective

All the patients involved in the programme were very enthusiastic in helping to develop and deliver training to tomorrow’s doctors. One of the individuals originally selected for the training could not be part of the training programme as she found it very difficult to provide structured feedback to the students. However, she was supported to attend the training sessions and continued to play an active part in developing the scenarios as well as sharing her experience with health professionals.

In the final debriefing session, all the actors reinforced the need for health professionals to be patient while communicating with people with intellectual disabilities. One person said, ‘We are not animals, we will not bite you. Please speak to us’. All actors reinforced the need for doctors to communicate with patients directly rather than with carers.

### Intellectual disabilities in the medical student curriculum

A survey of UK medical schools on the teaching offered on disability and rehabilitation concluded that the teaching was fragmented and inadequate.^13^ The survey recommended inclusion of disability and rehabilitation into clinical teaching. It highlighted the importance of emphasising functional assessment in teaching the physical examination and the wider use of standard assessment instruments. Although didactic lectures are a good method of teaching, it is equally important for medical students to have an opportunity to interact with people with intellectual disabilities, as the experience helps to create a positive image of people with intellectual disability in the minds of medical students.^14^

In a review of current research on human patient simulation in nursing education, Kameg *et al*^15^ stated that communication is an integral part of undergraduate education, with evidence that it improves health outcomes, patient adherence and patient satisfaction. Brenner^16^ has suggested that simulated patients can be helpful not only for exposing students to the variety of psychopathological states but also to teach and assess complex interpersonal processes such as empathic engagement and psychodynamic psychotherapy.

Various innovations have been introduced to improve the quality of training on intellectual disabilities provided to medical students in the UK. They include drama workshops run by a group of people with intellectual disabilities at St George’s Hospital Medical School in London, student-directed learning at the University of Dundee, and structured teaching programmes at the universities of Leeds and Edinburgh. May *et al*^17^ described a teaching programme that involved small groups of medical students working in partnership with people with intellectual disabilities on a specific task of mutual interest. At the end of the programme, it was noticed that the students had acquired greater respect for the abilities of people with intellectual disabilities and were more positively inclined towards them and their rights as citizens.

### Standardised patients: challenges

Although patient involvement is a useful tool in teaching medical students, there are certain issues that need to be taken into account. Tokenistic participation from patients and carers will be of little benefit to the participants and patient actors alike.^18^ The standardised patients should be empowered to be trainers by having a role in planning and delivery of training. It is essential that carers and patients who participate in training be on an equal footing with the moderators and professionals, because patients have a unique understanding of their illness and are best placed to judge trainees on their empathy and communication skills.^19^ In addition, the experience of being interviewed by medical students can generate anxiety and stress among the patient and proper support structures must be arranged to help them manage these feelings. This can also be achieved by having debriefing sessions involving professionals and actors wherein the actors can express their feelings and can help shape future training sessions.

Another important factor in involving service users as standardised patients is ensuring that the actors and their supporters are remunerated properly for their work. Local voluntary organisations or advocacy projects can prove to be a very useful link to make these arrangements.

### Future of champion trainers

The members of the standardised patient group have continued to meet regularly and have named themselves ‘champion trainers’. Because of the encouraging results and feedback, we aim to provide the communication skills and attitudes training programme to all medical students at UCL. This will be achieved by continuing to offer a special study module (SSM) on disability awareness and communication skills focusing on the issues relevant to intellectual disabilities. Other options include the provision of DVDs that can be uploaded to online teaching programmes. Finally, an extension to the training programme may be the development of similar training sessions for other health professionals and administration staff. Due to limited resources, we are planning to start an e-learning tool link, thereby increasing accessibility.
